# *catena*-Poly[[[aqua­(1,10-phenanthro­line)manganese(II)]-μ-adamantane-1,3-dicarboxyl­ato] monohydrate]

**DOI:** 10.1107/S1600536811041900

**Published:** 2011-10-12

**Authors:** Jian-Qiang Liu, Yun-Sheng Huang

**Affiliations:** a Guangdong Medical College, School of Pharmacy, Dongguan 523808, People’s Republic of China

## Abstract

In the title coordination polymer, {[Mn(C_12_H_14_O_4_)(C_12_H_8_N_2_)(H_2_O)]·H_2_O}_*n*_, the Mn^II^ atom has a highly distorted *cis*-MnN_2_O_4_ octa­hedral geometry arising from its coordination by a bidentate phenanthroline ligand, a water mol­ecule and monodentate and bidentate adamantane-1,3-dicarboxyl­ate dianions. The bridging dianion leads to [001] chains in the crystal. The chains are linked by O—H⋯O hydrogen bonds, involving both the coordinated and uncoordinated water mol­ecules, thereby forming a two-dimensional network.

## Related literature

For related structures, see: Liu & Wu (2010 ▶); Chen & Liu (2002 ▶). For background to the synthesis of functionalized adamantane compounds, see: Seidel & Stang (2002 ▶).
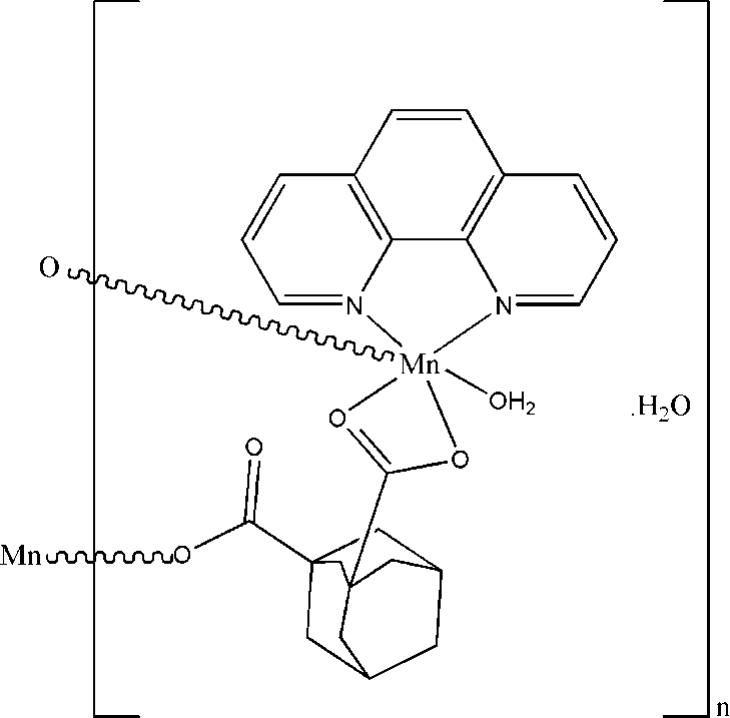


## Experimental

### 

#### Crystal data

[Mn(C_12_H_14_O_4_)(C_12_H_8_N_2_)(H_2_O)]·H_2_O
*M* *_r_* = 493.41
Monoclinic, 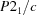

*a* = 13.248 (2) Å
*b* = 18.345 (3) Å
*c* = 9.3908 (17) Å
β = 105.283 (3)°
*V* = 2201.6 (6) Å^3^
*Z* = 4
Mo *K*α radiation
μ = 0.64 mm^−1^
*T* = 298 K
0.30 × 0.22 × 0.17 mm

#### Data collection

Bruker SMART CCD diffractometer
Absorption correction: multi-scan (*SADABS*; Bruker, 2008 ▶) *T*_min_ = 0.830, *T*_max_ = 0.898
10943 measured reflections
3918 independent reflections
2393 reflections with *I* > 2σ(*I*)
*R*_int_ = 0.046

#### Refinement

*R*[*F* ^2^ > 2σ(*F* ^2^)] = 0.040
*wR*(*F* ^2^) = 0.116
*S* = 0.83
3918 reflections
310 parameters
6 restraints
H atoms treated by a mixture of independent and constrained refinement
Δρ_max_ = 0.28 e Å^−3^
Δρ_min_ = −0.26 e Å^−3^

### 

Data collection: *SMART* (Bruker, 2008 ▶); cell refinement: *SAINT* (Bruker, 2008 ▶); data reduction: *SAINT*; program(s) used to solve structure: *SHELXS97* (Sheldrick, 2008 ▶); program(s) used to refine structure: *SHELXL97* (Sheldrick, 2008 ▶); molecular graphics: *ORTEP-3* (Farrugia, 1997 ▶); software used to prepare material for publication: *SHELXL97*.

## Supplementary Material

Crystal structure: contains datablock(s) I, global. DOI: 10.1107/S1600536811041900/hb6433sup1.cif

Structure factors: contains datablock(s) I. DOI: 10.1107/S1600536811041900/hb6433Isup2.hkl

Additional supplementary materials:  crystallographic information; 3D view; checkCIF report

## Figures and Tables

**Table:** 

Mn2—O3^i^	2.140 (2)
Mn2—O5	2.170 (2)
Mn2—O2	2.224 (2)
Mn2—N1	2.244 (2)
Mn2—O1	2.271 (2)
Mn2—N2	2.282 (2)

**Table:** 

O2—Mn2—O1	57.92 (7)

Symmetry code: (i) 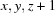.

**Table 2:** Hydrogen-bond geometry (Å, °)

*D*—H⋯*A*	*D*—H	H⋯*A*	*D*⋯*A*	*D*—H⋯*A*
O5—H5*WA*⋯O2^ii^	0.84 (1)	1.88 (1)	2.711 (3)	173 (5)
O5—H5*WB*⋯O4^i^	0.83 (1)	1.76 (1)	2.582 (3)	172 (5)
O6—H6*WA*⋯O3^iii^	0.84 (1)	2.60 (6)	3.062 (4)	116 (5)
O6—H6*WB*⋯O1^iv^	0.84 (1)	2.22 (4)	2.914 (4)	140 (5)

Symmetry codes: (i) 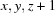; (ii) 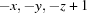; (iii) 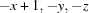; (iv) 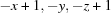.

**Fig. 1.**
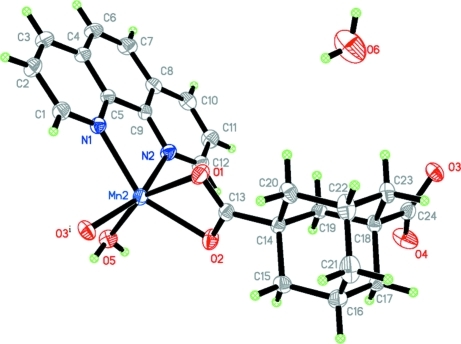
Molecular structure of (I), showing ellipsoids drawn at the 30% probability level. (symmetry code: (i): x, y, z-1).

**Fig. 2.**
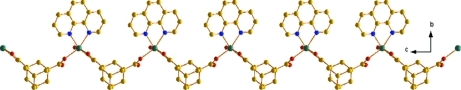
View of the 1D chain along the bc plane.

## supplementary crystallographic information

### Comment

Adamantane-1,3-dicarboxylate (H_2_L), is a dicarboxylaic acid and one of the most stable hydrocarbons, which was discovered in the 1930s. As a consequence of its stability, it can be produced catalytically from a wide various precursor organic substances (Seidel & Stang, 2002).  In our recent work, we have studied the supramolecular chemistry based on L and 2,2-bipy (Liu *et al.*, 2010). With this background in mind, we continued to our investigation and chose L as a bridging ligand and phenanthroline (phen) ligand to react with the d-block metal ions. Herein, we are interested in self-assembly reactions of Mn^II^ with H_2_L and phen, which led to the title compound, (I).

The title compound, {[Mn(L)(phen)(H_2_O)].H_2_O} is comprised of a Mn^II^,  one adamantane-1,3-dicarboxylate dianion and one phen ligand, one coordinated water molecule and one free water molecule. As illustrated in Fig. 1. the Mn^II^ has a highly distorted octahedral coordination sphere (Table 1) comprising two N atoms from one different phen ligand, three one oxygen atoms from the adjacent L ligands and one coordinated water molecule. In title compound, the Mn^II^ ions are linked by L ligands to form chains along the c axis (Fig. 2), and the resulting chains are further held together based on  O—H···O hydrogen bonds interactions, shaping 2D supramolecular sheet parallel to [010] (Table 2).

Compared to the title compound and {[Mn^II^(L)(2,2'-bipy).H_2_O]}n, the L exhibits bridging bidentate and chelated-bidentate modes in the latter compoud (Liu & Wu, 2010). Moreover,  a dinuclear unit Mn^II^ was also shaped due to the different coordinated mode. Thus, the assistant ligand could induce the separated fomration of structures (Chen & Liu., 2002).

### Experimental

A mixture of Mn(ac)_2_.H_2_O (25 mg, 0.1 mmol), H_2_L (21 mg, 0.1 mmol), phen (18 mg, 0.1 mmol), NaOH (0.1mmol) and 8 ml H_2_O and CH_3_OH (3ml) was stirred for 1h, and then the mixture was transferred to an 25-ml Teflon-lined reactor and kept under autogenous pressure at 435 K for 3 days,then cooled down to room temperature. Colourless blocks of (I) were obtained.

### Refinement

All H atoms attached to C and O (hydroxyl group) atoms were fixed geometrically and treated as riding with C—H = 0.93 Å  with U_iso_(H) = 1.2U_eq_(). H atoms of water molecules were located in a difference map and refined with restraints of O-H=0.83 (1)Å, and with U_iso_(H) = 1.5U_eq_(O).

### Crystal data

**Table:**

[Mn(C_12_H_14_O_4_)(C_12_H_8_N_2_)(H_2_O)]·H_2_O	*F*(000) = 1028
*M**_r_* = 493.41	*D*_x_ = 1.489 Mg m^−^^3^
Monoclinic, *P*2_1_/*c*	Mo *K*α radiation, λ = 0.71073 Å
Hall symbol: -P 2ybc	Cell parameters from 3918 reflections
*a* = 13.248 (2) Å	θ = 1.6–25.2°
*b* = 18.345 (3) Å	µ = 0.64 mm^−^^1^
*c* = 9.3908 (17) Å	*T* = 298 K
β = 105.283 (3)°	Block, colorless
*V* = 2201.6 (6) Å^3^	0.30 × 0.22 × 0.17 mm
*Z* = 4	

### Data collection

**Table:**

Bruker SMART CCD diffractometer	3918 independent reflections
Radiation source: fine-focus sealed tube	2393 reflections with *I* > 2σ(*I*)
graphite	*R*_int_ = 0.046
φ and ω scans	θ_max_ = 25.1°, θ_min_ = 1.6°
Absorption correction: multi-scan (*SADABS*; Bruker, 2008)	*h* = −15→13
*T*_min_ = 0.830, *T*_max_ = 0.898	*k* = −21→21
10943 measured reflections	*l* = −11→11

### Refinement

**Table:**

Refinement on *F*^2^	Primary atom site location: structure-invariant direct methods
Least-squares matrix: full	Secondary atom site location: difference Fourier map
*R*[*F*^2^ > 2σ(*F*^2^)] = 0.040	Hydrogen site location: inferred from neighbouring sites
*wR*(*F*^2^) = 0.116	H atoms treated by a mixture of independent and constrained refinement
*S* = 0.83	*w* = 1/[σ^2^(*F*_o_^2^) + (0.0752*P*)^2^ + 0.190*P*] where *P* = (*F*_o_^2^ + 2*F*_c_^2^)/3
3918 reflections	(Δ/σ)_max_ = 0.001
310 parameters	Δρ_max_ = 0.28 e Å^−^^3^
6 restraints	Δρ_min_ = −0.26 e Å^−^^3^

### Special details

**Table:**

Geometry. All esds (except the esd in the dihedral angle between two l.s. planes) are estimated using the full covariance matrix. The cell esds are taken into account individually in the estimation of esds in distances, angles and torsion angles; correlations between esds in cell parameters are only used when they are defined by crystal symmetry. An approximate (isotropic) treatment of cell esds is used for estimating esds involving l.s. planes.
Refinement. Refinement of F^2^ against ALL reflections. The weighted R-factor wR and goodness of fit S are based on F^2^, conventional R-factors R are based on F, with F set to zero for negative F^2^. The threshold expression of F^2^ > 2sigma(F^2^) is used only for calculating R-factors(gt) etc. and is not relevant to the choice of reflections for refinement. R-factors based on F^2^ are statistically about twice as large as those based on F, and R- factors based on ALL data will be even larger.

### Fractional atomic coordinates and isotropic or equivalent isotropic displacement parameters (Å^2^)

**Table:**

	*x*	*y*	*z*	*U*_iso_*/*U*_eq_	
Mn2	0.19018 (3)	0.04500 (3)	0.66751 (5)	0.03220 (17)	
O1	0.30979 (16)	0.00888 (12)	0.5476 (2)	0.0446 (6)	
O2	0.14940 (15)	−0.03061 (12)	0.4757 (2)	0.0417 (6)	
O3	0.24611 (15)	−0.02364 (12)	−0.1439 (2)	0.0402 (5)	
O4	0.09567 (17)	−0.06327 (14)	−0.1146 (2)	0.0623 (8)	
O5	0.04001 (16)	0.04967 (12)	0.7192 (2)	0.0415 (5)	
N1	0.28258 (17)	0.13908 (14)	0.7887 (2)	0.0325 (6)	
N2	0.13230 (18)	0.14711 (14)	0.5316 (2)	0.0356 (6)	
C19	0.2230 (2)	−0.04651 (16)	0.1959 (3)	0.0282 (6)	
H19A	0.2503	0.0023	0.1916	0.034*	
H19B	0.1478	−0.0429	0.1795	0.034*	
C5	0.2538 (2)	0.20657 (16)	0.7326 (3)	0.0309 (7)	
C9	0.1728 (2)	0.21121 (16)	0.5955 (3)	0.0314 (7)	
C14	0.2711 (2)	−0.07909 (16)	0.3488 (3)	0.0292 (7)	
C7	0.1847 (3)	0.34353 (18)	0.6131 (4)	0.0496 (9)	
H7	0.1618	0.3891	0.5744	0.060*	
C4	0.2981 (2)	0.27062 (18)	0.8028 (3)	0.0376 (8)	
C17	0.2031 (2)	−0.17043 (17)	0.0829 (3)	0.0370 (7)	
H17A	0.1278	−0.1674	0.0663	0.044*	
H17B	0.2177	−0.2013	0.0068	0.044*	
C1	0.3574 (2)	0.13474 (19)	0.9138 (3)	0.0395 (8)	
H1	0.3787	0.0888	0.9519	0.047*	
C10	0.0618 (2)	0.28050 (19)	0.4005 (3)	0.0440 (8)	
H10	0.0381	0.3247	0.3554	0.053*	
C13	0.2427 (2)	−0.03075 (16)	0.4641 (3)	0.0322 (7)	
C18	0.2479 (2)	−0.09385 (16)	0.0732 (3)	0.0300 (7)	
C8	0.1389 (2)	0.27964 (18)	0.5349 (3)	0.0376 (8)	
C23	0.3666 (2)	−0.09953 (18)	0.1002 (3)	0.0388 (8)	
H23A	0.3960	−0.0514	0.0953	0.047*	
H23B	0.3831	−0.1296	0.0245	0.047*	
C3	0.3762 (2)	0.2630 (2)	0.9355 (3)	0.0460 (9)	
H3	0.4080	0.3042	0.9858	0.055*	
C20	0.3902 (2)	−0.08430 (19)	0.3729 (3)	0.0393 (8)	
H20A	0.4221	−0.1045	0.4698	0.047*	
H20B	0.4190	−0.0360	0.3682	0.047*	
C15	0.2259 (2)	−0.15618 (16)	0.3527 (3)	0.0357 (7)	
H15A	0.2552	−0.1778	0.4490	0.043*	
H15B	0.1506	−0.1532	0.3364	0.043*	
C11	0.0218 (2)	0.2171 (2)	0.3362 (4)	0.0475 (9)	
H11	−0.0296	0.2173	0.2469	0.057*	
C24	0.1921 (2)	−0.05850 (17)	−0.0733 (3)	0.0354 (8)	
C21	0.3698 (3)	−0.20887 (19)	0.2604 (4)	0.0527 (10)	
H21A	0.4006	−0.2303	0.3565	0.063*	
H21B	0.3864	−0.2399	0.1860	0.063*	
C2	0.4056 (2)	0.19561 (19)	0.9908 (3)	0.0456 (9)	
H2	0.4573	0.1902	1.0789	0.055*	
C16	0.2516 (3)	−0.20380 (18)	0.2341 (3)	0.0448 (9)	
H16	0.2228	−0.2527	0.2382	0.054*	
C12	0.0587 (2)	0.15148 (19)	0.4056 (3)	0.0442 (8)	
H12	0.0299	0.1083	0.3607	0.053*	
C6	0.2598 (3)	0.33959 (18)	0.7406 (4)	0.0468 (9)	
H6	0.2873	0.3822	0.7891	0.056*	
C22	0.4146 (2)	−0.1333 (2)	0.2532 (3)	0.0441 (9)	
H22	0.4906	−0.1369	0.2697	0.053*	
O6	0.5446 (2)	0.0628 (2)	0.2049 (4)	0.0980 (11)	
H5WB	0.053 (4)	0.0136 (19)	0.775 (5)	0.147*	
H5WA	−0.016 (2)	0.046 (3)	0.654 (4)	0.147*	
H6WB	0.557 (4)	0.042 (3)	0.287 (3)	0.147*	
H6WA	0.595 (3)	0.090 (3)	0.200 (5)	0.147*	

### Atomic displacement parameters (Å^2^)

**Table:**

	*U*^11^	*U*^22^	*U*^33^	*U*^12^	*U*^13^	*U*^23^
Mn2	0.0366 (3)	0.0309 (3)	0.0281 (3)	0.0007 (2)	0.00675 (19)	−0.0015 (2)
O1	0.0418 (12)	0.0487 (15)	0.0423 (13)	−0.0064 (11)	0.0093 (10)	−0.0153 (11)
O2	0.0329 (12)	0.0563 (16)	0.0357 (12)	0.0018 (10)	0.0088 (9)	−0.0116 (11)
O3	0.0416 (12)	0.0472 (15)	0.0331 (12)	0.0018 (10)	0.0119 (10)	0.0123 (10)
O4	0.0420 (14)	0.085 (2)	0.0501 (15)	−0.0114 (13)	−0.0041 (11)	0.0307 (14)
O5	0.0350 (12)	0.0440 (15)	0.0434 (13)	0.0023 (11)	0.0068 (10)	0.0010 (11)
N1	0.0302 (13)	0.0357 (17)	0.0307 (14)	0.0010 (11)	0.0067 (10)	−0.0003 (12)
N2	0.0375 (14)	0.0339 (17)	0.0313 (14)	−0.0010 (12)	0.0019 (11)	0.0008 (12)
C19	0.0331 (15)	0.0214 (16)	0.0292 (15)	0.0023 (13)	0.0069 (12)	0.0021 (13)
C5	0.0326 (16)	0.0294 (19)	0.0325 (17)	−0.0018 (13)	0.0116 (13)	−0.0018 (14)
C9	0.0344 (16)	0.032 (2)	0.0291 (16)	0.0009 (14)	0.0096 (13)	−0.0039 (14)
C14	0.0332 (15)	0.0270 (18)	0.0272 (16)	0.0025 (13)	0.0078 (12)	−0.0025 (13)
C7	0.062 (2)	0.028 (2)	0.061 (2)	0.0025 (17)	0.0186 (19)	0.0050 (17)
C4	0.0433 (18)	0.031 (2)	0.0387 (19)	−0.0065 (15)	0.0123 (15)	−0.0047 (14)
C17	0.0471 (18)	0.0287 (18)	0.0351 (18)	0.0005 (15)	0.0106 (14)	−0.0053 (14)
C1	0.0360 (17)	0.043 (2)	0.0383 (18)	−0.0011 (15)	0.0069 (14)	−0.0005 (16)
C10	0.0435 (19)	0.043 (2)	0.046 (2)	0.0116 (17)	0.0123 (16)	0.0132 (17)
C13	0.0387 (18)	0.031 (2)	0.0242 (16)	0.0035 (14)	0.0032 (13)	0.0039 (13)
C18	0.0344 (16)	0.0311 (18)	0.0240 (15)	0.0037 (13)	0.0070 (12)	0.0035 (13)
C8	0.0377 (17)	0.037 (2)	0.0411 (19)	0.0030 (15)	0.0151 (15)	0.0053 (15)
C23	0.0404 (17)	0.047 (2)	0.0321 (17)	0.0041 (15)	0.0149 (14)	−0.0034 (15)
C3	0.047 (2)	0.044 (2)	0.047 (2)	−0.0128 (17)	0.0122 (16)	−0.0095 (17)
C20	0.0357 (17)	0.050 (2)	0.0297 (17)	0.0041 (15)	0.0043 (13)	0.0078 (16)
C15	0.0431 (18)	0.0290 (19)	0.0348 (17)	0.0050 (14)	0.0099 (14)	0.0082 (14)
C11	0.0414 (19)	0.055 (3)	0.042 (2)	0.0032 (17)	0.0031 (16)	0.0049 (18)
C24	0.0397 (18)	0.037 (2)	0.0271 (16)	−0.0004 (14)	0.0053 (14)	−0.0020 (14)
C21	0.068 (2)	0.047 (2)	0.044 (2)	0.0302 (19)	0.0156 (18)	0.0083 (17)
C2	0.0389 (18)	0.055 (3)	0.0370 (19)	−0.0057 (17)	−0.0006 (15)	−0.0042 (17)
C16	0.069 (2)	0.024 (2)	0.043 (2)	0.0035 (16)	0.0189 (17)	0.0021 (15)
C12	0.0486 (19)	0.043 (2)	0.0352 (19)	0.0000 (16)	0.0015 (15)	0.0011 (16)
C6	0.059 (2)	0.028 (2)	0.055 (2)	−0.0092 (16)	0.0175 (18)	−0.0052 (17)
C22	0.0324 (17)	0.064 (3)	0.0353 (18)	0.0188 (16)	0.0079 (14)	0.0039 (17)
O6	0.071 (2)	0.138 (4)	0.083 (2)	0.017 (2)	0.0171 (17)	0.043 (2)

### Geometric parameters (Å, °)

**Table:**

Mn2—O3^i^	2.140 (2)	C17—H17A	0.9700
Mn2—O5	2.170 (2)	C17—H17B	0.9700
Mn2—O2	2.224 (2)	C1—C2	1.390 (4)
Mn2—N1	2.244 (2)	C1—H1	0.9300
Mn2—O1	2.271 (2)	C10—C11	1.353 (5)
Mn2—N2	2.282 (2)	C10—C8	1.398 (4)
O1—C13	1.251 (3)	C10—H10	0.9300
O2—C13	1.269 (3)	C18—C24	1.524 (4)
O3—C24	1.269 (4)	C18—C23	1.529 (4)
O3—Mn2^ii^	2.140 (2)	C23—C22	1.539 (4)
O4—C24	1.236 (4)	C23—H23A	0.9700
O5—H5WB	0.833 (10)	C23—H23B	0.9700
O5—H5WA	0.835 (10)	C3—C2	1.358 (5)
N1—C1	1.325 (3)	C3—H3	0.9300
N1—C5	1.360 (4)	C20—C22	1.539 (4)
N2—C12	1.323 (3)	C20—H20A	0.9700
N2—C9	1.364 (4)	C20—H20B	0.9700
C19—C14	1.530 (4)	C15—C16	1.523 (4)
C19—C18	1.547 (4)	C15—H15A	0.9700
C19—H19A	0.9700	C15—H15B	0.9700
C19—H19B	0.9700	C11—C12	1.395 (4)
C5—C4	1.398 (4)	C11—H11	0.9300
C5—C9	1.445 (4)	C21—C22	1.517 (5)
C9—C8	1.402 (4)	C21—C16	1.522 (5)
C14—C13	1.521 (4)	C21—H21A	0.9700
C14—C20	1.536 (4)	C21—H21B	0.9700
C14—C15	1.540 (4)	C2—H2	0.9300
C7—C6	1.342 (4)	C16—H16	0.9800
C7—C8	1.431 (4)	C12—H12	0.9300
C7—H7	0.9300	C6—H6	0.9300
C4—C3	1.402 (4)	C22—H22	0.9800
C4—C6	1.429 (4)	O6—H6WB	0.839 (10)
C17—C16	1.524 (4)	O6—H6WA	0.844 (10)
C17—C18	1.537 (4)		
			
O3^i^—Mn2—O5	88.62 (8)	O2—C13—C14	119.4 (3)
O3^i^—Mn2—O2	105.10 (9)	C24—C18—C23	114.3 (2)
O5—Mn2—O2	99.46 (8)	C24—C18—C17	109.8 (2)
O3^i^—Mn2—N1	90.53 (9)	C23—C18—C17	108.9 (2)
O5—Mn2—N1	105.42 (8)	C24—C18—C19	106.6 (2)
O2—Mn2—N1	150.90 (8)	C23—C18—C19	109.1 (2)
O3^i^—Mn2—O1	95.98 (8)	C17—C18—C19	107.9 (2)
O5—Mn2—O1	157.34 (8)	C10—C8—C9	117.1 (3)
O2—Mn2—O1	57.92 (7)	C10—C8—C7	124.3 (3)
N1—Mn2—O1	96.73 (8)	C9—C8—C7	118.5 (3)
O3^i^—Mn2—N2	159.65 (9)	C18—C23—C22	109.6 (2)
O5—Mn2—N2	84.25 (8)	C18—C23—H23A	109.7
O2—Mn2—N2	94.88 (8)	C22—C23—H23A	109.7
N1—Mn2—N2	73.16 (9)	C18—C23—H23B	109.7
O1—Mn2—N2	97.92 (9)	C22—C23—H23B	109.7
C13—O1—Mn2	90.40 (18)	H23A—C23—H23B	108.2
C13—O2—Mn2	92.09 (17)	C2—C3—C4	120.0 (3)
C24—O3—Mn2^ii^	127.51 (19)	C2—C3—H3	120.0
Mn2—O5—H5WB	94 (4)	C4—C3—H3	120.0
Mn2—O5—H5WA	122 (4)	C14—C20—C22	109.5 (2)
H5WB—O5—H5WA	113 (3)	C14—C20—H20A	109.8
C1—N1—C5	117.8 (3)	C22—C20—H20A	109.8
C1—N1—Mn2	125.7 (2)	C14—C20—H20B	109.8
C5—N1—Mn2	116.35 (18)	C22—C20—H20B	109.8
C12—N2—C9	116.9 (3)	H20A—C20—H20B	108.2
C12—N2—Mn2	127.6 (2)	C16—C15—C14	110.3 (2)
C9—N2—Mn2	115.25 (18)	C16—C15—H15A	109.6
C14—C19—C18	111.2 (2)	C14—C15—H15A	109.6
C14—C19—H19A	109.4	C16—C15—H15B	109.6
C18—C19—H19A	109.4	C14—C15—H15B	109.6
C14—C19—H19B	109.4	H15A—C15—H15B	108.1
C18—C19—H19B	109.4	C10—C11—C12	119.0 (3)
H19A—C19—H19B	108.0	C10—C11—H11	120.5
N1—C5—C4	122.8 (3)	C12—C11—H11	120.5
N1—C5—C9	117.7 (3)	O4—C24—O3	123.4 (3)
C4—C5—C9	119.4 (3)	O4—C24—C18	117.9 (3)
N2—C9—C8	123.1 (3)	O3—C24—C18	118.6 (3)
N2—C9—C5	117.0 (3)	C22—C21—C16	109.6 (3)
C8—C9—C5	119.9 (3)	C22—C21—H21A	109.8
C13—C14—C19	108.7 (2)	C16—C21—H21A	109.8
C13—C14—C20	111.6 (2)	C22—C21—H21B	109.8
C19—C14—C20	108.6 (2)	C16—C21—H21B	109.8
C13—C14—C15	110.4 (2)	H21A—C21—H21B	108.2
C19—C14—C15	108.2 (2)	C3—C2—C1	119.1 (3)
C20—C14—C15	109.3 (2)	C3—C2—H2	120.4
C6—C7—C8	121.9 (3)	C1—C2—H2	120.4
C6—C7—H7	119.1	C21—C16—C15	109.6 (3)
C8—C7—H7	119.1	C21—C16—C17	109.8 (3)
C5—C4—C3	117.1 (3)	C15—C16—C17	109.3 (3)
C5—C4—C6	119.4 (3)	C21—C16—H16	109.4
C3—C4—C6	123.4 (3)	C15—C16—H16	109.4
C16—C17—C18	110.3 (2)	C17—C16—H16	109.4
C16—C17—H17A	109.6	N2—C12—C11	123.7 (3)
C18—C17—H17A	109.6	N2—C12—H12	118.1
C16—C17—H17B	109.6	C11—C12—H12	118.1
C18—C17—H17B	109.6	C7—C6—C4	120.8 (3)
H17A—C17—H17B	108.1	C7—C6—H6	119.6
N1—C1—C2	123.1 (3)	C4—C6—H6	119.6
N1—C1—H1	118.4	C21—C22—C20	110.0 (3)
C2—C1—H1	118.4	C21—C22—C23	109.8 (3)
C11—C10—C8	120.0 (3)	C20—C22—C23	109.4 (3)
C11—C10—H10	120.0	C21—C22—H22	109.2
C8—C10—H10	120.0	C20—C22—H22	109.2
O1—C13—O2	119.5 (3)	C23—C22—H22	109.2
O1—C13—C14	121.1 (3)	H6WB—O6—H6WA	111 (3)

Symmetry codes: (i) *x*, *y*, *z*+1; (ii) *x*, *y*, *z*−1.

### Hydrogen-bond geometry (Å, °)

**Table:**

*D*—H···*A*	*D*—H	H···*A*	*D*···*A*	*D*—H···*A*
O5—H5WA···O2^iii^	0.84 (1)	1.88 (1)	2.711 (3)	173 (5)
O5—H5WB···O4^i^	0.83 (1)	1.76 (1)	2.582 (3)	172 (5)
O6—H6WA···O3^iv^	0.84 (1)	2.60 (6)	3.062 (4)	116 (5)
O6—H6WB···O1^v^	0.84 (1)	2.22 (4)	2.914 (4)	140 (5)

Symmetry codes: (iii) −*x*, −*y*, −*z*+1; (i) *x*, *y*, *z*+1; (iv) −*x*+1, −*y*, −*z*; (v) −*x*+1, −*y*, −*z*+1.
